# Supplementary material for: Epidemiology of scrub typhus and other rickettsial infections (2018–22) in the hyper-endemic setting of Mizoram, North-East India
Source: PLoS Negl Trop Dis. 2023 Nov 1;17(11):e0011688. doi: 10.1371/journal.pntd.0011688 (PMC10642901; doi:10.1371/journal.pntd.0011688)
Supplement: S3 File — (DOCX) [file pntd.0011688.s003.docx]

**Supplementary File 3: Optimized Hotspots Analysis**

To map the hotspots of rickettsial infections in Mizoram, the Optimized Hotspot Analysis tool in ArcGIS Pro 3.0 software was used. We used all the available 836 case locations (villages/settlements) spread across Mizoram and the total number of cases in each location reported during 2018-2022 (**Supplementary File S2**). The hotspots were identified based on the Getis-Ord G_i_* statistic, using z-scores (standard deviations) and p-values, of each village/settlement within the context of the neighboring location. The very high/positive and very low/negative Z scores with significant p-values, populating the tails of the normal distribution curve are referred as hot and cold spots, respectively. Multiple significant levels (90%, 95%, and 99% confidence intervals) are used to report the clusters.

The Getis-Ord G_i_* statistics is given as,

$G_{i}^{*}=\frac{\sum_{j}^{n} w_{i,j} x_{j}-\overline{X}\sum_{j}^{n} w_{i,j}}{S\sqrt{\frac{\left[n\sum_{j=1}^{n} w_{_{i,j}}^{2}-\left(\sum_{j=1}^{n} w_{i,j}\right)^{2}\right]}{n-1}}}$………………………………………(1)

$\overline{X}=\frac{\sum_{j=1}^{n} x_{j}}{n}, S=\sqrt{\frac{\sum_{j=1}^{n} x_{_{j}}^{2}}{n}}-(\overline{X})^{2}$………………………………………(2)

where *x_j_* is the total cases recorded in a particular location *j* during 2018-2022, *w_i,j_* is the spatial weight between neighboring locations *i* and *j*, and *n* denote the total number of locations in Mizoram.

Randomization null hypothesis is the basis for statistical significance testing by the Optimized Hotspot Analysis tool. The z-scores and p-values are measures of statistical significance that denote whether the observed spatial clustering of high or low values are more pronounced than one would expect in a chance distribution of those same values. We assumed nearby neighboring case locations (within a fixed distance band) will have a larger influence on the computations for a target case location than case locations that are far away.

The Optimized Hotspot Analysis tool has aggregated incident data and adjusted results for multiple testing and spatial dependence based on the conceptualization of spatial relationships. In this study, based on the average spatial distribution of features, we fixed a minimum of 8 neighbors and an optimal fixed distance of 15 km radius as spatial constraints for the analysis.
