# Supplementary material for: Epidemiology of scrub typhus and other rickettsial infections (2018–22) in the hyper-endemic setting of Mizoram, North-East India
Source: PLoS Negl Trop Dis. 2023 Nov 1;17(11):e0011688. doi: 10.1371/journal.pntd.0011688 (PMC10642901; doi:10.1371/journal.pntd.0011688)
Supplement: S2 Table — (DOCX) [file pntd.0011688.s005.docx]

**S2 Table**

**Diagnostic tests based distribution of rickettsial infections across the districts of Mizoram (2018-22)**

| Districts | Tsutsugamushi test / Rapid immunochromatographic test |  | Weil Felix test | | | | | | | | | | |
| --- | --- | --- | --- | --- | --- | --- | --- | --- | --- | --- | --- | --- | --- |
|  | **Scrub typhus** |  | **Scrub typhus** |  | **Other rickettsal infections** | | | |  | **Mixed (scrub typhus and other rickettsial) infections** | | |  |
|  |  |  | **OXK** |  | **OX2** | **OX19** | **OX19 & OX2** | |  | **OXK & OX19** | **OXK & OX2** | **OXK, OX2& OX19** | **Total** |
|  | **13811** |  | **5838** |  | **1068** | **572** | **206** | |  | **331** | **466** | **620** | **9103** |
| 2018 | | | | | | | | | | | | | |
| Aizawl | 1008 |  | 2 |  | 0 | 0 | 0 | |  | 0 | 0 | 0 | 2 |
| Champhai | 34 |  | 0 |  | 0 | 0 | 0 | |  | 0 | 0 | 0 | 0 |
| Hnahthial | 27 |  | 0 |  | 0 | 0 | 0 | |  | 0 | 0 | 0 | 0 |
| Khawzawl | 9 |  | 0 |  | 0 | 0 | 0 | |  | 0 | 0 | 0 | 0 |
| Kolasib | 23 |  | 0 |  | 0 | 0 | 0 | |  | 0 | 0 | 0 | 0 |
| Lawngtlai | 119 |  | 0 |  | 0 | 0 | 0 | |  | 0 | 0 | 0 | 0 |
| Lunglei | 300 |  | 0 |  | 0 | 0 | 0 | |  | 0 | 0 | 0 | 0 |
| Mamit | 78 |  | 0 |  | 0 | 0 | 0 | |  | 0 | 0 | 0 | 0 |
| Saitual | 62 |  | 0 |  | 0 | 0 | 0 | |  | 0 | 0 | 0 | 0 |
| Serchhip | 513 |  | 0 |  | 0 | 0 | 0 | |  | 0 | 0 | 0 | 0 |
| Siaha | 8 |  | 0 |  | 0 | 0 | 0 | |  | 0 | 0 | 0 | 0 |
|  | **2181 (15.79%)** |  | **2** |  | **0** | **0** | **0** | |  | **0** | **0** | **0** | **2 (0.02%)** |
| 2019 | | | | | | | | | | | | | |
| Aizawl | 3729 |  | 150 |  | 6 | 5 | 0 | |  | 0 | 2 | 82 | 245 |
| Champhai | 15 |  | 17 |  | 44 | 19 | 3 | |  | 1 | 0 | 0 | 84 |
| Hnahthial | - |  | 3 |  | 104 | 4 | 3 | |  | 0 | 11 | 2 | 127 |
| Khawzawl | 10 |  | 11 |  | 97 | 135 | 21 | |  | 1 | 0 | 0 | 265 |
| Kolasib | - |  | 55 |  | 10 | 5 | 12 | |  | 5 | 6 | 4 | 97 |
| Lawngtlai | 125 |  | 0 |  | 0 | 0 | 0 | |  | 0 | 15 | 0 | 15 |
| Lunglei | 59 |  | 0 |  | 0 | 0 | 0 | |  | 0 | 0 | 0 | 0 |
| Mamit | 106 |  | 0 |  | 0 | 0 | 0 | |  | 0 | 0 | 12 | 12 |
| Saitual | 259 |  | 0 |  | 0 | 0 | 0 | |  | 0 | 0 | 0 | 0 |
| Serchhip | 1204 |  | 61 |  | 0 | 1 | 0 | |  | 0 | 0 | 0 | 62 |
| Siaha | 55 |  | 0 |  | 2 | 1 | 0 | |  | 0 | 0 | 0 | 3 |
|  | **5562 (40.27%)** |  | **297** |  | **263** | **170** | **39** | |  | **7** | **34** | **100** | **910 (10.00%)** |
| 2020 | | | | | | | | | | | | | |
| Aizawl | 1341 |  | 407 |  | 0 | 1 | | 2 |  | 1 | 17 | 196 | 624 |
| Champhai | 7 |  | 30 |  | 35 | 8 | | 0 |  | 1 | 0 | 2 | 76 |
| Hnahthial | - |  | 4 |  | 142 | 3 | | 1 |  | 1 | 5 | 0 | 156 |
| Khawzawl | - |  | 47 |  | 16 | 110 | | 11 |  | 8 | 10 | 1 | 203 |
| Kolasib | 2 |  | 12 |  | 38 | 7 | | 26 |  | 4 | 20 | 5 | 112 |
| Lawngtlai | 286 |  | 0 |  | 0 | 0 | | 0 |  | 0 | 48 | 0 | 48 |
| Lunglei | 136 |  | 0 |  | 68 | 2 | | 1 |  | 0 | 0 | 0 | 71 |
| Mamit | 21 |  | 186 |  | 86 | 53 | | 8 |  | 14 | 2 | 12 | 361 |
| Saitual | 41 |  | 38 |  | 0 | 0 | | 0 |  | 0 | 0 | 0 | 38 |
| Serchhip | 124 |  | 10 |  | 0 | 0 | | 0 |  | 0 | 0 | 0 | 10 |
| Siaha | 47 |  | 5 |  | 4 | 4 | | 0 |  | 0 | 0 | 0 | 13 |
|  | **2005 (14.52%)** |  | **739** |  | **389** | **188** | | **49** |  | **29** | **102** | **216** | **1712 (18.81%)** |
| 2021 | | | | | | | | | | | | | |
| Aizawl | 819 |  | 473 |  | 79 | 18 | | 6 |  | 4 | 50 | 99 | 729 |
| Champhai | 14 |  | 37 |  | 30 | 6 | | 5 |  | 7 | 2 | 2 | 89 |
| Hnahthial | - |  | 17 |  | 40 | 4 | | 2 |  | 1 | 19 | 0 | 83 |
| Khawzawl | - |  | 11 |  | 6 | 7 | | 0 |  | 0 | 2 | 0 | 26 |
| Kolasib | - |  | 34 |  | 36 | 18 | | 13 |  | 2 | 24 | 2 | 129 |
| Lawngtlai | 186 |  | 1 |  | 0 | 0 | | 0 |  | 82 | 17 | 0 | 100 |
| Lunglei | 169 |  | 8 |  | 57 | 1 | | 0 |  | 0 | 0 | 0 | 66 |
| Mamit | 34 |  | 66 |  | 11 | 19 | | 1 |  | 22 | 8 | 2 | 129 |
| Saitual | 2 |  | 20 |  | 39 | 11 | | 2 |  | 2 | 16 | 0 | 90 |
| Serchhip | 126 |  | 202 |  | 11 | 7 | | 1 |  | 1 | 2 | 1 | 225 |
| Siaha | 103 |  | 1 |  | 0 | 0 | | 0 |  | 0 | 0 | 0 | 1 |
|  | **1453 (10.52%)** |  | **870** |  | **309** | **91** | | **30** |  | **121** | **140** | **106** | **1667 (18.31%)** |
| 2022 | | | | | | | | | | | | | |
| Aizawl | 1021 |  | 913 |  | 10 | 11 | | 11 |  | 29 | 14 | 74 | 1062 |
| Champhai | 145 |  | 108 |  | 6 | 6 | | 2 |  | 7 | 4 | 4 | 137 |
| Hnahthial | 38 |  | 442 |  | 25 | 14 | | 8 |  | 14 | 46 | 9 | 558 |
| Khawzawl | 5 |  | 360 |  | 19 | 21 | | 9 |  | 12 | 22 | 20 | 463 |
| Kolasib | 62 |  | 271 |  | 6 | 12 | | 8 |  | 12 | 12 | 1 | 322 |
| Lawngtlai | 500 |  | 140 |  | 1 | 5 | | 4 |  | 20 | 6 | 8 | 184 |
| Lunglei | 371 |  | 82 |  | 6 | 3 | | 5 |  | 6 | 8 | 5 | 115 |
| Mamit | 26 |  | 565 |  | 12 | 30 | | 16 |  | 35 | 30 | 39 | 727 |
| Saitual | 27 |  | 445 |  | 7 | 10 | | 14 |  | 13 | 20 | 15 | 524 |
| Serchhip | 137 |  | 598 |  | 15 | 11 | | 11 |  | 26 | 28 | 22 | 711 |
| Siaha | 278 |  | 8 |  | 0 | 0 | | 0 |  | 0 | 0 | 1 | 9 |
|  | **2610 (18.90%)** |  | **3932** |  | **107** | **123** | | **88** |  | **174** | **190** | **198** | **4812 (52.86%)** |
