# Supplementary material for: Epidemiology of scrub typhus and other rickettsial infections (2018–22) in the hyper-endemic setting of Mizoram, North-East India
Source: PLoS Negl Trop Dis. 2023 Nov 1;17(11):e0011688. doi: 10.1371/journal.pntd.0011688 (PMC10642901; doi:10.1371/journal.pntd.0011688)
Supplement: S3 Table — (DOCX) [file pntd.0011688.s006.docx]

**S3 Table**

**Incidence rates of scrub typhus, other rickettsial and mixed infections across the districts of Mizoram (2018-2022)**

| Cases / 1000 persons-year | Aizawl | | Champhai | Hnahthial | Khawzawl | Kolasib | Lawngtlai | Lunglei | Mamit | Saitual | Serchhip | Siaha | Mizoram |
| --- | --- | --- | --- | --- | --- | --- | --- | --- | --- | --- | --- | --- | --- |
| Population size, 2020 estimates* | 457119 | | 103556 | 35759 | 37261 | 108625 | 147897 | 138290 | 97973 | 57217 | 63688 | 47185 | 1294570 |
| Scrub typhus |  | |  |  |  |  |  |  |  |  |  |  |  |
| 2018 | 2.21 | | 0.33 | 0.76 | 0.24 | 0.21 | 0.80 | 2.17 | 0.80 | 1.08 | 8.05 | 0.17 | 1.69 |
| 2019 | 8.49 | | 0.31 | 0.08 | 0.56 | 0.51 | 0.85 | 0.43 | 1.08 | 4.53 | 19.86 | 1.17 | 4.53 |
| 2020 | 3.82 | | 0.36 | 0.11 | 1.26 | 0.13 | 1.93 | 0.98 | 2.11 | 1.38 | 2.10 | 1.10 | 2.12 |
| 2021 | 2.83 | | 0.49 | 0.48 | 0.30 | 0.31 | 1.26 | 1.28 | 1.02 | 0.38 | 5.15 | 2.20 | 1.79 |
| 2022 | 4.23 | | 2.44 | 13.42 | 9.80 | 3.07 | 4.33 | 3.28 | 6.03 | 8.25 | 11.54 | 6.06 | 5.05 |
| Avg. incidence rate  (95% CI) | 4.32  (4.23-4.40) | | 0.79  (0.71-0.86) | 2.97  (2.72-3.22) | 2.43  (2.21-2.66) | 0.85  (0.77-0.92) | 1.84  (1.74-1.93) | 1.63  (1.53-1.72) | 2.21  (2.08-2.34) | 3.12  (2.92-3.33) | 9.34  (9.01-9.68) | 2.14  (1.95-2.33) | 3.04  (2.99-3.08) |
| Other rickettsial infections | |  |  |  |  |  |  |  |  |  |  |  |  |
| 2018 | - | | - | - | - | - | - | - | - | - | - | - | - |
| 2019 | 0.02 | | 0.64 | 3.10 | 6.79 | 0.25 | 0.00 | 0.00 | 0.00 | 0.00 | 0.02 | 0.06 | 0.36 |
| 2020 | 0.01 | | 0.42 | 4.08 | 3.68 | 0.65 | 0.00 | 0.51 | 1.50 | 0.00 | 0.00 | 0.17 | 0.01 |
| 2021 | 0.02 | | 0.04 | 0.13 | 0.03 | 0.06 | 0.00 | 0.04 | 0.03 | 0.09 | 0.03 | 0.00 | 0.03 |
| 2022 | 0.07 | | 0.14 | 1.31 | 1.32 | 0.24 | 0.07 | 0.10 | 0.59 | 0.54 | 0.58 | 0.00 | 0.25 |
| Avg. incidence rate  (95% CI) | 0.08  (0.07-0.09) | | 0.40  (0.34-0.46) | 2.45  (2.19-2.70) | 3.03  (2.75-3.31) | 0.44  (0.38-0.50) | 0.02  (0.01-0.03) | 0.26  (0.22-0.30) | 0.60  (0.53-0.68) | 0.36  (0.28-0.44) | 0.22  (0.17-0.28) | 0.06  (0.02-0.09) | 0.36  (0.34-0.37) |
| Mixed (scrub typhus and other rickettsial) infections | | |  |  |  |  |  |  |  |  |  |  |  |
| 2018 | - | | - | - | - | - | - | - | - | - | - | - | - |
| 2019 | 0.18 | | 0.01 | 0.36 | 0.03 | 0.14 | 0.10 | 0.00 | 0.12 | 0.00 | 0.00 | 0.00 | 0.11 |
| 2020 | 0.47 | | 0.03 | 0.17 | 0.51 | 0.27 | 0.32 | 0.00 | 0.29 | 0.00 | 0.00 | 0.00 | 0.27 |
| 2021 | 0.33 | | 0.11 | 0.56 | 0.05 | 0.26 | 0.67 | 0.00 | 0.33 | 0.31 | 0.06 | 0.00 | 0.28 |
| 2022 | 0.26 | | 0.14 | 1.93 | 1.45 | 0.23 | 0.23 | 0.14 | 1.06 | 0.84 | 1.19 | 0.02 | 0.43 |
| Avg. incidence rate  (95% CI) | 0.31  (0.29-0.34) | | 0.07  (0.05-0.10) | 0.76  (0.61-0.90) | 0.51  (0.40-0.62) | 0.22  (0.18-0.27) | 0.33  (0.28-0.38) | 0.03  (0.02-0.05) | 0.45  (0.38-0.52) | 0.29  (0.22-0.36) | 0.31  (0.25-0.38) | 0.01  (-0.01-0.02) | 0.27  (0.26-0.29) |

*Source: Wang W, Kim R, Subramanian SV. Population Estimates for Districts and Parliamentary Constituencies in India, 2020. V1 ed: Harvard Dataverse; 2021.
