# Supplementary material for: Epidemiology of scrub typhus and other rickettsial infections (2018–22) in the hyper-endemic setting of Mizoram, North-East India
Source: PLoS Negl Trop Dis. 2023 Nov 1;17(11):e0011688. doi: 10.1371/journal.pntd.0011688 (PMC10642901; doi:10.1371/journal.pntd.0011688)
Supplement: S4 Table — (DOCX) [file pntd.0011688.s007.docx]

**S4 Table**

**Incidence rates of rickettsial infections across districts of Mizoram based on antigenic strains (2019-2022)**

| Cases/1000 persons-year | Aizawl | Champhai | Hnahthial | Khawzawl | Kolasib | Lawngtlai | Lunglei | Mamit | Saitual | Serchhip | Siaha | Mizoram |
| --- | --- | --- | --- | --- | --- | --- | --- | --- | --- | --- | --- | --- |
|  |  |  |  |  |  |  |  |  |  |  |  |  |
| OXK* |  |  |  |  |  |  |  |  |  |  |  |  |
| 2019 | 0.33 | 0.16 | 0.08 | 0.30 | 0.51 | 0.00 | 0.00 | 0.00 | 0.00 | 0.96 | 0.00 | 0.23 |
| 2020 | 0.89 | 0.29 | 0.11 | 1.26 | 0.11 | 0.00 | 0.00 | 1.90 | 0.66 | 0.16 | 0.11 | 0.57 |
| 2021 | 1.03 | 0.36 | 0.48 | 0.30 | 0.31 | 0.01 | 0.06 | 0.67 | 0.35 | 3.17 | 0.02 | 0.67 |
| 2022 | 2.00 | 1.04 | 12.36 | 9.66 | 2.49 | 0.95 | 0.59 | 5.77 | 7.78 | 9.39 | 0.17 | 3.04 |
| Avg. incidence rate  (95% CI) | 1.06  (1.02-1.11) | 0.46  (0.40-0.53) | 3.26  (2.96-3.55) | 2.88  (2.61-3.15) | 0.86  (0.77-0.94) | 0.24  (0.20-0.28) | 0.16  (0.13-0.20) | 2.08  (1.94-2.23) | 2.20  (2.01-2.39) | 3.42  (3.19-3.65) | 0.07  (0.04-0.11) | 1.13  (1.10-1.16) |
| OX2 |  |  |  |  |  |  |  |  |  |  |  |  |
| 2019 | 0.01 | 0.42 | 2.91 | 2.60 | 0.09 | 0.00 | 0.00 | 0.00 | 0.00 | 0.00 | 0.04 | 0.20 |
| 2020 | 0 | 0.34 | 3.97 | 0.43 | 0.35 | 0 | 0.49 | 0.88 | 0 | 0 | 0.08 | 0.30 |
| 2021 | 0.17 | 0.29 | 1.12 | 0.16 | 0.33 | 0.00 | 0.41 | 0.11 | 0.68 | 0.17 | 0.00 | 0.24 |
| 2022 | 0.02 | 0.06 | 0.70 | 0.51 | 0.06 | 0.01 | 0.04 | 0.12 | 0.12 | 0.24 | 0.00 | 0.08 |
| Avg. incidence rate  (95% CI) | 0.05  (0.04-0.06) | 0.28  (0.23-0.33) | 2.17  (1.93-2.42) | 0.93  (0.77-1.08) | 0.21  (0.16-0.25) | 0.00  (0.00-0.01) | 0.24  (0.20-0.28) | 0.28  (0.23-0.33) | 0.20  (0.14-0.26) | 0.10  (0.06-0.14) | 0.03  (0.01-0.06) | 0.21  (0.19-0.22) |
| OX19 |  |  |  |  |  |  |  |  |  |  |  |  |
| 2019 | 0.01 | 0.18 | 0.11 | 3.62 | 0.05 | 0.00 | 0.00 | 0.00 | 0.00 | 0.02 | 0.02 | 0.13 |
| 2020 | 0.00 | 0.08 | 0.08 | 2.95 | 0.06 | 0.00 | 0.01 | 0.54 | 0.00 | 0.00 | 0.08 | 0.15 |
| 2021 | 0.04 | 0.06 | 0.11 | 0.19 | 0.17 | 0.00 | 0.01 | 0.19 | 0.19 | 0.11 | 0.00 | 0.07 |
| 2022 | 0.02 | 0.06 | 0.39 | 0.56 | 0.11 | 0.03 | 0.02 | 0.31 | 0.17 | 0.17 | 0.00 | 0.10 |
| Avg. incidence rate  (95% CI) | 0.02  (0.01-0.03) | 0.09  (0.06-0.12) | 0.17  (0.11-0.24) | 1.83  (1.61-2.05) | 0.10  (0.07-0.13) | 0.01  (0.00-0.02) | 0.01  (0.00-0.02) | 0.26  (0.21-0.31) | 0.09  (0.05-0.13) | 0.07  (0.04-0.11) | 0.03  (0.00-0.05) | 0.11  (0.10-0.12) |
| OXK and OX2 |  |  |  |  |  |  |  |  |  |  |  |  |
| 2019 | 0.00 | 0.00 | 0.31 | 0.00 | 0.06 | 0.10 | 0.00 | 0.00 | 0.00 | 0.00 | 0.00 | 0.03 |
| 2020 | 0.04 | 0.00 | 0.14 | 0.27 | 0.18 | 0.32 | 0.00 | 0.02 | 0.00 | 0.00 | 0.00 | 0.08 |
| 2021 | 0.11 | 0.02 | 0.53 | 0.05 | 0.22 | 0.11 | 0.00 | 0.08 | 0.28 | 0.03 | 0.00 | 0.11 |
| 2022 | 0.03 | 0.04 | 1.29 | 0.59 | 0.11 | 0.04 | 0.06 | 0.31 | 0.35 | 0.44 | 0.00 | 0.15 |
| Avg. incidence rate  (95% CI) | 0.05  (0.04-0.06) | 0.01  (0.00-0.03) | 0.57  (0.44-0.69) | 0.23  (0.15-0.30) | 0.14  (0.11-0.18) | 0.15  (0.11-0.18) | 0.01  (0.00-0.02) | 0.10  (0.07-0.13) | 0.16  (0.11-0.21) | 0.12  (0.08-0.16) | 0.00  (0.00-0.00) | 0.09  (0.08-0.10) |
| OXK and OX19 |  |  |  |  |  |  |  |  |  |  |  |  |
| 2019 | 0.00 | 0.01 | 0.00 | 0.03 | 0.05 | 0.00 | 0.00 | 0.00 | 0.00 | 0.00 | 0.00 | 0.01 |
| 2020 | 0.00 | 0.01 | 0.03 | 0.21 | 0.04 | 0.00 | 0.00 | 0.14 | 0.00 | 0.00 | 0.00 | 0.02 |
| 2021 | 0.01 | 0.07 | 0.03 | 0.00 | 0.02 | 0.55 | 0.00 | 0.22 | 0.03 | 0.02 | 0.00 | 0.09 |
| 2022 | 0.06 | 0.07 | 0.39 | 0.32 | 0.11 | 0.14 | 0.04 | 0.36 | 0.23 | 0.41 | 0.00 | 0.13 |
| Avg. incidence rate  (95% CI) | 0.02  (0.01-0.02) | 0.04  (0.02-0.06) | 0.11  (0.06-0.17) | 0.14  (0.08-0.20) | 0.05  (0.03-0.07) | 0.17  (0.14-0.21) | 0.01  (0.00-0.02) | 0.18  (0.14-0.22) | 0.07  (0.03-0.10) | 0.11  (0.07-0.15) | 0.00  (0.00-0.00) | 0.06  (0.06-0.07) |
| OX2 and OX19 |  |  |  |  |  |  |  |  |  |  |  |  |
| 2019 | 0 | 0.03 | 0.08 | 0.56 | 0.11 | 0.00 | 0.00 | 0.00 | 0.00 | 0.00 | 0.00 | 0.03 |
| 2020 | 0.00 | 0.00 | 0.03 | 0.30 | 0.24 | 0.00 | 0.01 | 0.08 | 0.00 | 0.00 | 0.00 | 0.04 |
| 2021 | 0.01 | 0.05 | 0.06 | 0.00 | 0.12 | 0.00 | 0.00 | 0.01 | 0.03 | 0.02 | 0.00 | 0.02 |
| 2022 | 0.02 | 0.02 | 0.22 | 0.24 | 0.07 | 0.03 | 0.04 | 0.16 | 0.24 | 0.17 | 0.00 | 0.07 |
| Avg. incidence rate  (95% CI) | 0.01  (0.01-0.02) | 0.02  (0.01-0.04) | 0.10  (0.05-0.15) | 0.28  (0.19-0.36) | 0.14  (0.10-0.17) | 0.01  (0.00-0.01) | 0.01  (0.00-0.02) | 0.06  (0.04-0.09) | 0.07  (0.04-0.10) | 0.05  (0.02-0.07) | 0.00  (0.00-0.00) | 0.04  (0.03-0.05) |
| OXK, OX2 and OX19 |  |  |  |  |  |  |  |  |  |  |  |  |
| 2019 | 0.18 | 0.00 | 0.06 | 0.00 | 0.04 | 0.00 | 0.00 | 0.12 | 0.00 | 0.00 | 0.00 | 0.08 |
| 2020 | 0.43 | 0.02 | 0.00 | 0.03 | 0.05 | 0.00 | 0.00 | 0.12 | 0.00 | 0.00 | 0.00 | 0.17 |
| 2021 | 0.22 | 0.02 | 0.00 | 0.00 | 0.02 | 0.00 | 0.00 | 0.02 | 0.00 | 0.02 | 0.00 | 0.08 |
| 2022 | 0.16 | 0.04 | 0.25 | 0.54 | 0.01 | 0.05 | 0.04 | 0.40 | 0.26 | 0.35 | 0.02 | 0.15 |
| Avg. incidence rate  (95% CI) | 0.25  (0.22-0.27) | 0.02  (0.01-0.03) | 0.08  (0.03-0.12) | 0.14  (0.08-0.20) | 0.03  (0.01-0.04) | 0.01  (0.00-0.02) | 0.01  (0.00-0.02) | 0.17  (0.13-0.21) | 0.07  (0.03-0.10) | 0.09  (0.05-0.13) | 0.01  (-0.01-0.02) | 0.12  (0.11-0.13) |

*In 2018, only two OXK positive cases were reported and hence not included.
