# Supplementary material for: Epidemiology of scrub typhus and other rickettsial infections (2018–22) in the hyper-endemic setting of Mizoram, North-East India
Source: PLoS Negl Trop Dis. 2023 Nov 1;17(11):e0011688. doi: 10.1371/journal.pntd.0011688 (PMC10642901; doi:10.1371/journal.pntd.0011688)
Supplement: S6 Table — (DOCX) [file pntd.0011688.s009.docx]

**S6 Table**

**Distribution of rickettsial cases across different occupational groups in Mizoram (2018-2022)**

| Occupation | Scrub typhus | | Other infections | | | | | | Mixed infections | | | | | | Total (%) |
| --- | --- | --- | --- | --- | --- | --- | --- | --- | --- | --- | --- | --- | --- | --- | --- |
|  |  |  | **OX2** | | **OX19** | | **OX2 and OX19** | | **OXK and OX2** | | **OXK and OX19** | | **OXK, OX2 and OX 19** | |  |
|  | Cases (%) | | Cases (%) | | Cases (%) | | Cases (%) | | Cases (%) | | Cases (%) | | Cases (%) | |  |
| Business | 1955 (9.9) | | 64 (6.0) | | 23 (4.0) | | 22 (10.7) | | 38 (8.2) | | 23 (6.9) | | 63 (10.2) | | 2188 (9.5) |
| Construction workers | 1104 (5.6) | | 94 (8.8) | | 9 (1.6) | | 8 (3.9) | | 28 (6.0) | | 13 (3.9) | | 29 (4.7) | | 1285 (5.6) |
| Farmer | 9805 (49.9) | | 672 (62.9) | | 283 (49.5) | | 93 (45.1) | | 282 (60.5) | | 171 (51.7) | | 318 (51.3) | | 11624 (50.7) |
| Government services | 1344 (6.8) | | 56 (5.2) | | 44 (7.7) | | 15 (7.3) | | 22 (4.7) | | 15 (4.5) | | 54 (8.7) | | 1550 (6.8) |
| Pre school | 968 (4.9) | | 16 (1.5) | | 18 (3.1) | | 13 (6.3) | | 13 (2.8) | | 25 (7.6) | | 26 (4.2) | | 1079 (4.7) |
| Students | 4301(21.9) | | 155 (14.5) | | 193 (33.7) | | 54 (26.2) | | 76 (16.3) | | 78 (23.6) | | 125 (20.2) | | 4982 (21.7) |
| Others (aged 71 and above) | 174 (0.9) | | 11 (1.0) | | 2 (0.3) | | 1 (0.5) | | 7 (1.5) | | 6 (1.8) | | 5 (0.8) | | 206 (0.9) |
|  | **19651** |  | **1068** |  | **572** |  | **206** |  | **466** |  | **331** |  | **620** |  | **22914** |
