# Supplementary figures and images for: Epidemiology of scrub typhus and other rickettsial infections (2018–22) in the hyper-endemic setting of Mizoram, North-East India
Source: PLoS Negl Trop Dis. 2023 Nov 1;17(11):e0011688. doi: 10.1371/journal.pntd.0011688 (PMC10642901; doi:10.1371/journal.pntd.0011688)

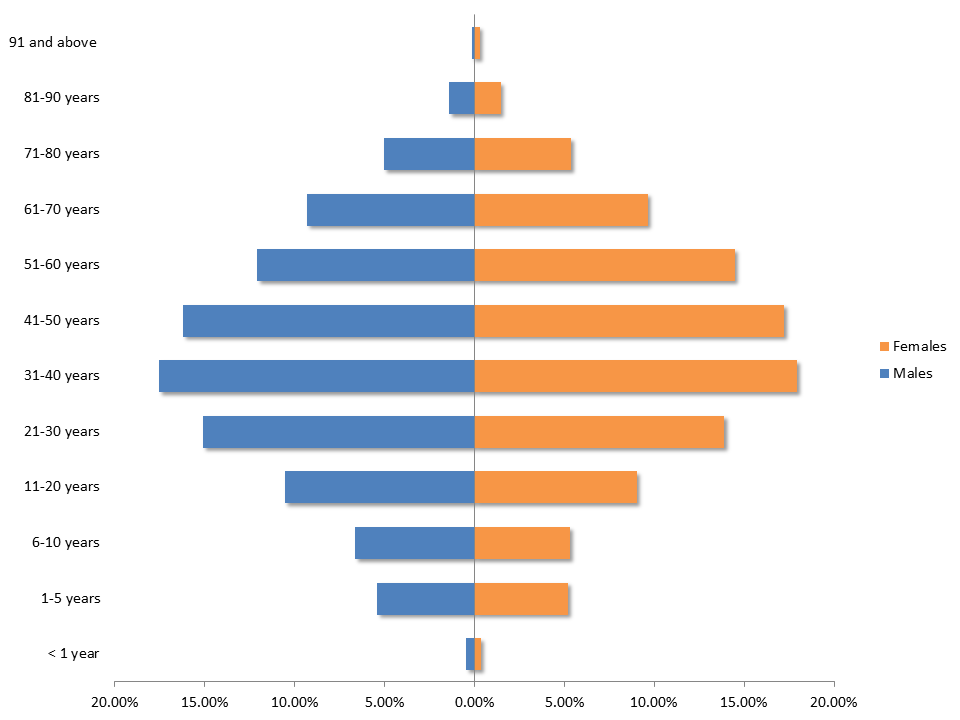

Supplement: S1 Fig — (TIFF) [file pntd.0011688.s015.tiff]
