# Supplementary material for: Epidemiology of scrub typhus and other rickettsial infections (2018–22) in the hyper-endemic setting of Mizoram, North-East India
Source: PLoS Negl Trop Dis. 2023 Nov 1;17(11):e0011688. doi: 10.1371/journal.pntd.0011688 (PMC10642901; doi:10.1371/journal.pntd.0011688)
Supplement: Attachment — Submitted filename: Answers to reviewers queries.docx [file pntd.0011688.s016.docx]

On behalf of all the authors, I profusely thank the reviewers and editors for spending their valuable time and efforts to improve this manuscript. We have incorporated most of the suggestions, and we strongly feel the manuscript is much stronger after the edits.

Our point-by-point reply to the queries are given below and italicized.

**Key Review Criteria Required for Acceptance**
As you describe the new analyses required for acceptance, please consider the following:

**Methods**
-Are the objectives of the study clearly articulated with a clear testable hypothesis stated?
-Is the study design appropriate to address the stated objectives?
-Is the population clearly described and appropriate for the hypothesis being tested?
-Is the sample size sufficient to ensure adequate power to address the hypothesis being tested?
-Were correct statistical analysis used to support conclusions?
-Are there concerns about ethical or regulatory requirements being met?

Reviewer #1: Yes

Reviewer #2:

**-Are the objectives of the study clearly articulated with a clear testable hypothesis stated?** Yes

**-Is the study design appropriate to address the stated objectives? No, The prevalence of each region (county/city) is affected by the population ratio and number of patients. Spatial mapping and GIS analysis are preferably described by incidence rates.**

**-Is the population clearly described and appropriate for the hypothesis being tested? No. Same answer as previous question**

*As the testing units [PHCs, CHCs, district and private hospitals (line no. 127)] recorded only the positive cases, the study used incidence rates to describe the prevalence. And as suggested, we have included a new GIS analysis based on the incidence rates (Figure 2).*

*The study considered districts population based on projected population estimates, 2020, retrieved from Harvard Dataverse (*[*https://doi.org/10.7910/DVN/RXYJR6*](https://doi.org/10.7910/DVN/RXYJR6)*). The reference is cited in line no. 158.*

**-Is the sample size sufficient to ensure adequate power to address the hypothesis being tested? Yes
-Were correct statistical analysis used to support conclusions? Yes
-Are there concerns about ethical or regulatory requirements being met? No**

**Results**
-Does the analysis presented match the analysis plan?
-Are the results clearly and completely presented?
-Are the figures (Tables, Images) of sufficient quality for clarity?

Reviewer #1: Yes

Reviewer #2:

**-Does the analysis presented match the analysis plan? Yes
-Are the results clearly and completely presented? No, In table 3, the adjusted odds ratio of death in Districts are preferably analyzed by case fatal rate calculated per 1000 persons.**

**-Are the figures (Tables, Images) of sufficient quality for clarity? No, they need to be revised as comments above.**

*The odds ratio was calculated using the rickettsial disease outcome (dependent variable)-recovery or death (line nos. 149-151). The details of CFRs (calculated per 1000 persons) are provided in the Supplementary Table S10 (line numbers 290 and 291).*

**Conclusions**
-Are the conclusions supported by the data presented?
-Are the limitations of analysis clearly described?
-Do the authors discuss how these data can be helpful to advance our understanding of the topic under study?
-Is public health relevance addressed?

Reviewer #1: Yes.

Reviewer #2:

-Are the conclusions supported by the data presented? Yes
-Are the limitations of analysis clearly described? Yes
-Do the authors discuss how these data can be helpful to advance our understanding of the topic under study? Yes
-Is public health relevance addressed? Yes

**Editorial and Data Presentation Modifications?**
Use  this section for editorial suggestions as well as relatively minor modifications of existing data that would enhance clarity. If the only modifications needed are minor and/or editorial, you may wish to recommend “Minor Revision” or “Accept”.
Reviewer #1: -

Reviewer #2:

**-It is better to indicate references in the sentences on page 5, lines 106 to 108.**

*Reference for lines 106 to 108: Lalmalsawma P, Pautu L. Scenario of Scrub typhus disease in Mizoram, Northeast India. International Journal of Current Advanced Research. 2017;6:6341-4. The reference is added in the revised manuscript.*

**-In methods, The positive cut-off of the Weil-Felix test should be described.**

*The positive cut-off of the Weil-Felix test is 1:160 and above. We have added the cut-off value in line no. 139.*

**-In Table 1, it would be useful to describe the differences in the diagnosis test of scrub typhus between 2018 and after 2018. In particular, in 2018, it would be better to describe only the diagnosis of scrub typhus.**

*Thank you for the suggestion. We have incorporated the details in Supplementary Table 2.*

**The spatial distribution information will be of more value if the terrain in those provinces is explained.**

*The terrain of these districts have been detailed in a supplementary table in our recent manuscript “Lalmalsawma P, Balasubramani K, James MM, Pautu L, Sarma DK and Balabaskaran Nina P. Malaria hotspots and climate change trends in the hyper-endemic malaria settings of Mizoram along the India-Bangladesh borders.* ***Sci Rep*** *13, 4538, 2023****.***

*We have added the following line in the revised manuscript “The district-wise geographic and socio-demographic variables have been provided in our earlier study (Reference)” [line no. 173].*

S**ummary and General Comments**
Use  this section to provide overall comments, discuss strengths/weaknesses of the study, novelty, significance, general execution and scholarship. You may also include additional comments for the author, including concerns about dual publication, research ethics, or publication ethics. If requesting major revision, please articulate the new experiments that are needed.

Reviewer #1: The research paper by Vanramliana et al. (PNTD-D-23-00849, Epidemiology of scrub typhus and other rickettsial infections (2018-22) in the hyperendemic setting of Mizoram, North-East India) provides the results of screening and line listing of scrub typhus and related infections in Mizoram. They say that low fatality of 0.35 as against ~6% from other locations could be used as a template for disease monitoring and management.

**Specific points:**

**“line-listed data positive for” – the authors should provide information on the total number of different screening tests done, year-wise and region-wise. That will provide the % of positive cases.**

*It is an important suggestion. However, as of now, the testing units across Mizoram (PHCs, CHCs, district and private hospitals) do not provide information on the total number of individuals screened for rickettsial diseases, and how many of them were positive (% positive); the line listing data provide only data on the number of positive cases year and district-wise. Even though we could not calculate the % positivity, the incidence rate calculated based on the total positive cases in each district provides important insight into the transmission dynamics of scrub typhus and other rickettsial diseases in Mizoram.*

*In the future, we have requested IDSP to record the details of the various screening tests done in the state to quantify the % of rickettsial positivity in febrile patients attending the clinic by Weil-Felix or rapid tsutsugamushi test.*

**“836 case locations were identified” – it is unclear if authors found coordinates for only 836 cases or 836 distinct coordinates were found. How many cases could not be mapped? They may include coordinate information in S1 file.**

*These 836 coordinates are villages/settlement locations across Mizoram. As suggested, we have added these locations along with the case number as an additional Supplementary file (S2). As these locations are very remote and the coordinates could be updated in the future, we have provided only the names of the case locations and not the coordinates.*

**Table 1 p-value – for which column or row and what test?**

*A column with information on the infection (scrub typhus or other rickettsial infections or mixed infections) for which individuals were positive was created and was cross tabulated (chi-square) with each of the independent variables. P-values were tabulated.*

**Table 2 – how incident rate range was calculated? Give year-wise population size for those places in supplemental.**

*The references used for incidence rate range calculation has been added in the revised manuscript (Line no. 157). The population size has been added to the Supplementary Table S3.*

*References for incidence rate range calculation:* [*https://www.health.pa.gov/topics/HealthStatistics/Statistical-Resources/UnderstandingHealthStats/Documents/Confidence_Intervals_for_a_Crude_Rate.pdf*](https://www.health.pa.gov/topics/HealthStatistics/Statistical-Resources/UnderstandingHealthStats/Documents/Confidence_Intervals_for_a_Crude_Rate.pdf)

**Is there data/information on severity of the disease and length to recovery? Also, what decides hospitalization after screening? These data might be important for disease management and might influence the outcome/death. No such info is given in the S1 file.**

*We greatly appreciate these important queries. This information would have provided important insight into the pathogenesis and disease outcome. Unfortunately, this information is not required in the line listing proforma that doctors (across Mizoram) have to enter/fill. Considering the importance of this information, we have requested the Integrated Disease Surveillance Program (IDSP) to include this information in the line listing proforma from now on. We have included this as a limitation in the study (Line nos. 380-382).*

**“76.54% of the hospitalized rickettsial cases suffered death” – seems an erroneous statement. That will be 3000+ deaths! Check.**

*For clarity, we have reframed the sentence as follows “Of the 81 rickettsial related deaths occurred, 76.54% (n=62) were hospitalised cases” (Line no. 299).*

**Reviewer #2: The strengths of the article lie in the structure and epidemiological descriptions. However, the data analysis (esp, Spatial Mapping and GIS analysis) is incomplete. The map should provide incidence rates instead of total cases in each regions.**
*As suggested, we have included a new GIS analysis based on the incidence rates (Figure 2).*
