# Supplementary material for: Epidemiology of scrub typhus and other rickettsial infections (2018–22) in the hyper-endemic setting of Mizoram, North-East India
Source: PLoS Negl Trop Dis. 2023 Nov 1;17(11):e0011688. doi: 10.1371/journal.pntd.0011688 (PMC10642901; doi:10.1371/journal.pntd.0011688)
Supplement: Attachment — Submitted filename: Response to reviewers.docx [file pntd.0011688.s017.docx]

On behalf of all the authors, I profusely thank the reviewers and editors for spending their valuable time and efforts to improve this manuscript. We have incorporated the suggestion, and we strongly feel the manuscript is much stronger after the edits.

Our reply to the query is given below and italicized.

**Query**:

**Still the authors have to address reviewer 1 queries. Please address comprehensively to the queries, particularly how you assess and incorporate spatial correlation into the models.**

*We used the line listing data of scrub typhus and other rickettsial infections across Mizoram to carry out the statistical analysis.*

*We ran a multivariate binary logistic regression model to assess the simultaneous effect of independent variables (age, sex, place of residence, occupation, eschar, and hospitalization) on the dependent variable—scrub typhus outcome (death or recovery).*

*The model met all the logistic regression assumptions—non-linear association between predictor (independent) and response (dependent) variables, no issue of multicollinearity between independent variables (*variance inflation factor*, VIF <1.005), dichotomous nature of the dependent variable, and mutually exclusive categories. The fitted model was further evaluated for parameters like Wald statistic and goodness of fit statistics (Hosmer and Lemeshow test), and was then used to estimate the scrub typhus outcome, given the risk factors.*

*The study has not considered any spatial parameter for the regression model/analysis. We have employed the hotspot analysis independently (based on case locations) just to illustrate the spatial distribution of the cases/clusters. We would like to clarify that we have not considered these spatial distributions in the regression model. We have included a new supplementary file (****S3 File****) to provide a better understanding of how the hotspot analysis was carried out,*
